# The association of *XRCC1 *gene single nucleotide polymorphisms with response to neoadjuvant chemotherapy in locally advanced cervical carcinoma

**DOI:** 10.1186/1756-9966-28-91

**Published:** 2009-06-29

**Authors:** Xiao-Dong Cheng, Wei-Guo Lu, Feng Ye, Xiao-Yun Wan, Xing Xie

**Affiliations:** 1 Department of Gynecologic Oncology and Women's Reproductive Health Key Laboratory of Zhejiang Province, Women's Hospital, School of Medicine, Zhejiang University, Hangzhou, PR China

## Abstract

**Background:**

Platinum-based neoadjuvant chemotherapy (NAC) is new therapeutic strategy for locally advanced cervical carcinoma, but the variables used to predict NAC response are still infrequently reported. The aim of our study was to investigate the association between *XRCC1 *gene single nucleotide polymorphisms (SNPs) and NAC response.

**Methods:**

Seventy patients with locally advanced cervical carcinoma who underwent NAC were collected. SNPs of *XRCC1 *(at codon 194 and 399) and XRCC1 protein expression were detected. The association of *XRCC1 *gene SNPs and protein expression with NAC response were analyzed.

**Results:**

Response to NAC was not statistically significant in three genotypes, Arg/Arg, Arg/Trp, Trp/Trp of *XRCC1 *at codon 194(X^2 ^= 1.243, P = 0.07), while responses were significantly different in genotypes Arg/Arg, Arg/Gln, Gln/Gln of *XRCC1 *at codon 399 (X^2 ^= 2.283, P = 0.020). The risk of failure to chemotherapy in the patients with a Gln allele(Arg/Gln+Gln/Gln) was significantly greater than that with Arg/Arg(OR = 3.254, 95%CI 1.708 ~ 14.951). The expression level of XRCC1 protein was significantly associated with response to NAC. Moreover, the genotype with the Gln allele(Arg/Gln+Gln/Gln) at codon 399, but not codon at 194, presented a significantly higher level of XRCC1 protein expression than that with Arg/Arg genotype (F = 2.699, p = 0.009).

**Conclusion:**

SNP of *XRCC1 *gene at codon 399 influences the response of cervical carcinoma to platinum-based NAC. This is probably due to changes in expression of XRCC1 protein, affecting response to chemotherapy.

## Background

Cervical carcinoma is the second most common malignancy, and continues to be a leading cause of cancer death in women. It is generally accepted that radical surgery or radiotherapy can be curative for the majority of patients with early-stage cervical carcinoma. However, the prognosis of locally advanced or bulky disease remains very poor, and the optimal management for those patients is still a matter of debate, new therapeutic strategies, such as neoadjuvant chemotherapy (NAC) and concurrent chemoradiation, have been adopted to improve the prognosis for those patients [1].

Many clinical studies have revealed that NAC is highly effective for patients with locally advanced cervical carcinoma, the use of NAC followed by radical surgery and/or radiation for the treatment of cervical carcinoma has been investigated extensively in the past decade, it has been reported that NAC with cisplatinum-based chemotherapeutic regimens have high response rates (ranging from 53% to 94%) [1,2]. However, those who have a poor response to chemotherapy usually fail to respond to radiotherapy, and have a poor prognosis. Thus, NAC may delay definitive treatment, increase cost, and result in poorer outcomes in those patients [3]. It is important to select appropriate patients before undergoing NAC; however, the variables used to predict NAC response are infrequently reported in locally advanced cervical carcinoma.

Cisplatin is considered to be the most effective drug for the treatment of cervical carcinoma, and usually is an essential element in the NAC regimen, but the mechanisms dictating variable response to chemotherapy among individuals are still unknown. Because platinum compounds produce adducts and breaks in the DNA double helix, individual variability of DNA repair may be relevant in modulating the efficacy of such cytotoxic agents. In resent years, some studies have shown that the molecular condition of DNA repair genes can predict the response of chemotherapy in some human cancers [4]. The presence of single-nucleotide polymorphisms (SNPs) among patients suggests that genetic variability may contribute to variations in responsiveness to chemotherapy [5].

X-ray repair cross-complementing gene 1 (*XRCC1*) is one of the most important DNA repair genes. The XRCC1 protein physically interacts with ligase III and poly(ADP-robose) polymerase, acting as a scaffold in the removal of adducts through both single-strand break repair and base excision repair (BER), and in the repair of other types of cisplatin-induced damage, including double-strand breaks, through a nonhomologous end-joining pathway [6]. There are three main coding polymorphisms in the *XRCC1 *gene: at codon 194 (Arg to Trp), 280 (Arg to His), and 399 (Arg to Gln). It was suggested that SNPs in the *XRCC1 *gene may alter the ability of *XRCC1 *to repair damaged DNA, especially SNPs at codon 399 [7]. Some studies have shown that genetic polymorphisms of the *XRCC1 *gene are associated with response to platinum-based chemotherapy in non-small-cell lung cancer, colorectal cancer, and breast cancer [8,9], but few studies have investigated the association of *XRCC1 *SNPs with response to chemotherapy in locally advanced cervical carcinoma. Only one study has analyzed *XRCC1 *SNPs at codon 399, and another study has analyzed SNPs at codon 194 recently, the results have shown that the *XRCC1 *Arg399Trp polymorphism or the *XRCC1 *Arg194Trp polymorphism is associated with the response to platinum-based NAC in cervical cancer, but the number of cases were all small (36 patients and 66 patients respectively) [10,11]. No results of this two SNPs in the same patients were showed.

To clarify the influence of the *XRCC1 *gene polymorphisms on the response to NAC, in the present study, we examined the association of the different genotypes (at codons 194 and 399), as well as protein expression with NAC response in patients with locally advanced cervical carcinoma.

## Methods

### Patient enrollment

From June 2003 to June 2007, a total of 109 patients with histologically confirmed locally advanced cervical carcinoma (FIGO stage IB2-IIA at least 4 cm in diameter) underwent NAC and subsequent radical hysterectomy in Women's Hospital School of Medicine, Zhejiang University. Of those, 70 patients who had complete clinical data, peripheral blood samples, and cervical carcinoma tissures by biopsy just before chemotherapy were enrolled in the study. Each patient signed a form to indicate informed consent before chemotherapy.

### Chemotherapy

NAC regimens consisted of cisplatinum-based combined chemotherapy. The regimens included BVP (blemycin 15 mg/m^2^, on d1, d7; cisplatin 60 mg/m^2 ^on d1; vindesine 4 mg/m^2 ^on d1–d2) in 47 patients, BIP (blemycin 15 mg/m^2 ^on d1; ifosfamide 1 g/m^2 ^on d1–d5; cisplatin 50 mg/m^2 ^on d1) in 15 patients, TP (taxol 60 mg/m^2 ^d1; cisplatin 60 mg/m^2 ^on d1) in 8 patients. NAC was administered every 3 to 4 weeks, for one to three cycles: one cycle in 15 patients, two cycles in 49 patients, and three cycles in 6 patients. All of the chemotherapeutic agents were administered intravenously.

### Evaluation of chemotherapy response

The chemotherapy response was evaluated two weeks after completion of the final cycle according to WHO criteria, if no obvious response occurred after two cycles, the patient would not accept another cycle of chemotherapy. Tumor size was measured by pelvic examination and colposcopy as the product of the maximal perpendicular diameter of the tumor. complete response (CR) indicates disappearance of the disease, partial response (PR) indicates at least 50% reduction in tumor load, stable disease (SD) indicates that the lesion showed ≤ 25% progression or <50% shrinkage, and progression of disease (PD) indicates >25% enlargement of the lesion, or appearance of a new lesion. CR and PR were considered to be a good response; SD and PD, a poor response.

### DNA extraction

Genomic DNA was extracted from peripheral blood lymphocytes by the routine phenol/chloroform method. First, white blood cells were separated from red blood cells by washing three times in phosphate buffer solution. Then, the DNA was extracted with phenol/chloroform and was precipitated with cold ethanol. All DNA samples were dissolved in water and stored at -20°C.

### Genotyping

The two SNPs were detected using modified polymerase chain reaction (PCR) mismatch amplification (MA-PCR). The two forward primers for *XRCC1 *gene Arg194Trp site were 5'-GGGGGCTCTCTTCTTCAGGC-3' and 5'-GGGGGCTCTCTTCTTCAGGT-3', which differ in the last base; the reverse primer was 5'-CGCTGGCTGTGACTATGAAG-3', which together produce a 362 bp fragment. The two forward primers for the *XRCC1 *gene Arg399Gln site were 5'-CGTCGGCGGCTGCCCTCCTG-3' and 5'-CGTCGGCGGCTGCCCTCCTA-3'; the reverse primer was 5'-TTACAGGCGTGAGCCACTGC-3', which together produce a 354 bp fragment. For assessing the reproducibility of results, all samples were tested twice by different technical personnel and the results were concordant for all masked duplicate sets.

### Detection of protein expression

#### Primary Antibodies

The rabbit anti-human polyclonal antibodies specific for XRCC1 were purchased from Santa Cruz Biotechnology™, Inc, Santa Cruz, California, USA.

#### Immunohistochemistry and Evaluation

XRCC1 protein expression was detected by Immunohistochemistry, using the EnVision two-step method. The cervical carcinoma samples from patients were obtained from the paraffin-embedded tissue blocks from cervical biopsy before therapy.

The quantitative immunoreactive scores (H-Score method) were used to evaluate the results, calculated by Σp(i+1), with i representing the various levels of stain: 0, no detectable stain in the nucleus or cytoplasm; 1, yellowish stain; 2, yellow stain; 3, brown stain; p represented the percentage of samples of each stain level. Five random fields (400× objective) were counted, and slides were reviewed independently by two pathologists without knowledge of the clinical data, The average of the the quantitative immunohistochemical scores data was calculated as the final result for each sample.

### Statistical analysis

Difference in frequencies of the *XRCC1 *genotypes and alleles between the different chemotherapy response groups were evaluated by *X*^2 ^test and Fisher's test. The association between *XRCC1 *polymorphisms and protein expression were evaluated by variance analysis. We also evaluated the observed genotype frequencies with those calculated from the Hardy-Weinberg equilibrium equation (p^2^+2pq+q^2 ^= 1, where p is the frequency of the variant allele and q = 1-p). We applied logistic regression to calculate odds ratios (ORs) and 95% confidence intervals (95% CI) for the association between the genotypes and the risk of chemotherapy failure(SD or PD). The variance analysis was used for measurement data. All *P*-values were two-tailed and values < 0.05 were considered statistically significant. Statistical package for social science software (Version 11.5, SPSS Inc, Chicago, IL) was used to perform all of the statistical analysis.

## Results

### Response of NAC

In the total of 70 patients, NAC response was as follows: CR in 2 patients, PR in 58 patients, and SD in 10 patients. No PD was found. Accordingly, the good response rate was 85.71%; the poor response rate was 14.39%.

### *XRCC1 *allele and genotype frequencies

The allele frequencies of *XRCC1 *194Arg(C) and 194Trp(T) were 65.8% and 34.2%, respectively in all patients; the allele frequencies of *XRCC1 *399Arg (G) and 399Gln (A) were 80.1% and 19.9%, respectively. The distributions of these genotype frequencies were all in agreement with those expected from the Hardy-Weinberg equilibrium model, the Hardy-Weinberg equilibrium test showed *X*^2 ^= 0.03 and *X*^2 ^= 1.62 respectively.

### The association between *XRCC1 *polymorphisms and response to NAC

Results are shown in Table 1 for the analysis of NAC response of patients with different genotypes. The NAC good response rate (CR+PR) among patients with locally advanced cervical carcinoma who carry three different homozygous genotypes at codon 194 [Arg/Arg (CC), Arg/Trp (CT), and Trp/Trp(TT)] were 82.35%, 100%, and 66.7% respectively. No statistically significant differences were found among polymorphisms of *XRCC1 *at codon 194 (*X*^2 ^= 1.243, *P *= 0.07).

**Table 1:** The association between *XRCC1 *polymorphisms at codons 194 and 399 and NAC response in locally advanced cervical carcinoma

*XRCC1 *genotype	N	Good response [N (%)]	Poor response [N (%)]	OR	95%CI
Codon 194 Arg/Arg	34	28 (82.35)	6 (17.65)		
Arg/Trp	24	24 (100)	0 (0)		
Trp/Trp	12	8 (66.67)	4 (33.33)	2.333	0.52~10.35
Arg/Trp+ Trp/Trp	36	32 (88.89)	4 (11.11)	0.583*	0.14~2.28
Codon 399 Arg/Arg	44	40(90.90)	4 (9.10)		
Arg/Gln	2	0 (0)	2 (100)		
Gln/Gln	24	20 (83.33)	4 (16.67)	2.000	0.452 ~8.842
Arg/Gln+ Gln/Gln	26	20 (76.92)	6 (23.08)	3.254**	1.708 ~ 14.951

Good response: CR+PR; Poor response: SD+PD; OR: odds ratio

*: Arg/Trp+Trp/Trp vs Arg/Arg; **: Arg/Gln+Gln/Gln vs Arg/Arg

*XRCC1 *gene polymorphisms at codon 399 were found to be significantly associated with NAC response. The NAC response rate (CR+PR) among patients with locally advanced cervical carcinoma carrying three different homozygous genotypes at codon 399 [Arg/Arg (GG), Arg/Gln (GA), and Gln/Gln(AA)] were 90.0%, 0% (0/2), and 83.33%, respectively (*X*^2 ^= 2.283, *P *= 0.02). Logistic regression analysis showed a significantly increased rate of failure of NAC in patients with at least one Gln allele [Arg/Gln(GA)+Gln/Gln(AA)] versus the Arg/Arg (GG) genotype (odds ratio 3.254; 95% CI 1.708–14.951; *P *= 0.002).

### The association between XRCC1 protein expression and NAC response

The level of XRCC1 protein expression was significantly higher in patients with poor response to NAC (SD+PD) than it was in those with good response (CR+PR) (2.99 ± 0.38 vs. 1.94 ± 0.28; *t *= 13.64, *P *= 0.008).

### The association between *XRCC1 *polymorphisms and protein expression

The association of the variant genotypes at codon 194 and 399 with expression of the XRCC1 protein in locally advanced cervical carcinoma tissues were further evaluated, as shown in Table 2. No statistically significant difference was found between the codon 194 polymorphism and XRCC1 protein expression(F = 1.186, *P *= 0.103); however, there was a statistically significant association between codon 399 polymorphism and XRCC1 protein expression (F = 15.915, *P *< 0.001).

**Table 2:** The association between *XRCC1 *polymorphisms and protein expression in locally advanced cervical carcinoma

*XRCC1 *genotype	N	X ± SD	F	*P*
Codon 194				
Arg/Arg	34	2.306 ± 0.658		
Arg/Trp	24	1.813 ± 0.341	1.186	0.103
Trp/Trp	12	2.217 ± 0.446		
Codon 399				
Arg/Arg	44	1.986 ± 0.404		
Arg/Gln	24	2.224 ± 0.604	15.915	<0.001
Gln/Gln	2	3.890 ± 0.000		
Arg/Gln + Gln/Gln	26	2.352 ± 0.735	2.699 *	0.009

*: Arg/Gln+Gln/Gln vs Arg/Arg

In addition, the level of expression of XRCC1 protein in patients with at least one Gln allele [Arg/Gln (GA) + Gln/Gln (AA)] was significantly higher than that in the patients with the Arg/Arg (GG) genotype (F = 2.699, *P *= 0.009).

## Discussion

It is well known that DNA repair is very important in the maintenance of genetic stability, and in protection against the initiation of cancer. Owing to its possible effects on gene expression, polymorphisms of DNA repair genes related to metabolism may influence tumor response to chemotherapy or radiotherapy. The identification of molecular variables that predict either sensitivity or resistance to chemotherapy is of major interest in selecting the first-line treatment most likely to be effective. Because *XRCC1 *is one of the most important DNA repair genes, the main aim of the present study was to determine whether the *XRCC1 *genetic polymorphisms could predict clinical response of patients with locally advanced cervical carcinoma to platinum-based NAC.

Some studies have assessed the association between *XRCC1 *gene polymorphisms and chemotherapy response in various carcinomas, but the results are inconsistent. There has been increasing evidence that decreased DNA repair capacity resulting from genetic polymorphisms of various DNA repair genes is associated with improved survival of cancer patients treated with platinum-based chemotherapy, especially in non-small cell lung cancer [12]. Studies addressing the association of *XRCC1 *gene polymorphisms at codon 194 with chemotherapy response have focused mainly on non-small cell lung cancer. Wang and his colleagues reported 105 patients with non-small lung cancer undergoing platinum-based chemotherapy, and found that the response rate was significantly higher in patients carrying at least one Trp allele than in those with the Arg/Arg genotype (43.1% vs. 20.3%) [13]. In patients with advanced-stage lung cancer, the risk of failure of chemotherapy was five-fold higher in patients with Arg/Arg genotype at codon 194 than in those with the Trp/Trp genotype [14]. On the other hand, some other studies did not find that the SNPs of *XRCC1 *contributed to susceptibility to cancer or to sensitivity to chemotherapy. These inconsistent results may be related to the different types of cancers studied in different ethnic populations [15,16].

Only one study assessed the association between XRCC1 gene polymorphisms at codon 194 and NAC response in cervical cancer, recently, Kim and his colleagues reported 66 patients with cervical cancer undergoing platinum-based NAC, the results showed that the genotypes of XRCC1 Arg194Trp was associated with the response [11]. But Our current report did not find any significant association, the inconsistent results may be related to the different ethnic populations and the limitatiom of the sample.

It has been suggested that the SNPs of *XRCC1 *at codon 399 may influence the outcome of cisplatinum-based chemotherapy in some human carcinomas, but the results are also variable. Wang and his colleagues reported that in patients with non-small cell lung cancer who received the platinum-based chemotherapy, the response rate was significantly higher in patients with the Arg/Arg genotype than that in those with at least one Gln allele (41.5% vs. 21.2%). In contrast, other studies of patients with neck cancer revealed that sensitivity to chemotherapy was higher in patients with a Gln allele than in those with other genotypes [13,17]. Moreno and colleagues also found that the prognosis of colorectal cancer patients receiving chemotherapy with 5-FU was better in patients with the 399Gln/Gln genotype than in those with Arg/Arg or Arg/Gln genotype [18]. While in a recent study, no significant association was found between the SNPs of *XRCC1 *at codon 399 and the response to chemotherapy in non-small cell lung cancer [14].

Our study showed that the response to chemotherapy in locally advanced cervical carcinoma was significantly higher in patients with the Arg/Arg genotype at codon 399 than in those with the Arg/Gln or Gln/Gln genotype (90.0% vs. 76.92%). The risk of failure of NAC therapy was 3.254 fold higher in patients carrying at least one Gln allele compared with those carrying no Gln allele. Our findings suggest that SNPs of the *XRCC1 *gene at codon 399 influences the response of cervical carcinoma to platinum-based neoadjuvant chemotherapy, and that the genotype carrying at least one Gln allele may be considered to be a candidate molecular marker to predict poor response to NAC in locally advanced cervical carcinoma.

The fact that SNPs of *XRCC1 *at codon 399 influences response to NAC in locally advanced cervical carcinoma affirms previous results reported by the studies of other carcinomas, but the exact mechanism remains unknown [13,17,19]. Some studies have shown that resistance to platinum-based agents was related to the overexpression of DNA-repair protein [20]. Dabholkar and colleagues found that the mRNA level of some DNA repair gene was significantly increased in platinum-resistant ovarian carcinoma, indicating that the level of DNA repair gene expression correlates with the response to platinum-based chemotherapy [21].

Similarly, our results also showed that the level of XRCC1 protein expression was significantly higher in patients with poor response than in those with good response to NAC in locally advanced cervical carcinoma. In addition, we found that this altered expression of the XRCC1 protein was associated with *XRCC1 *genotype variation at codon 399, the protein expression was significantly higher in the patients with a Gln allele (Arg/Gln or Gln/Gln) than that with the Arg/Arg genotype in locally advanced cervical carcinoma. Our findings suggest that the genotype with at least one Gln allele probably increases the expression of XRCC1 protein, and consequently, results in poor response to platinum-based chemotherapy in patients with locally advanced cervical carcinoma. To our knowledge, this is the first investigation of *XRCC1 *gene SNPs, protein expression, and their association with response to chemotherapy. Further study is needed to clarify the mechanism behind this phenomenon.

We have demonstrated that SNPs of the *XRCC1 *gene at codon 399 influence the response of patients with locally advanced cervical carcinoma to platinum-based NAC. Patients with a genotype carrying at least one Gln allele have an increased risk of failure to respond to chemotherapy compared with those carrying no Gln allele. This reduced response to chemotherapy is probably due to elevated expression of XRCC1 protein in those patients who have at least one Gln allele.

## Competing interests

The authors declare that they have no competing interests.

## Authors' contributions

XDC have made substantial contributions to conception, and drafting the manuscript. WGL have made substantial contributions to patients sample collection. FY carried out the molecular genetic studies. XYW carried out the protein expression detection and performed the statistical analysis. XX conceived of the study, and participated in its design, and coordination and helped to draft the manuscript. All authors read and approved the final manuscript.
